# Effects of prenatal transportation stress on liver gene expression in male and female Brahman calves

**DOI:** 10.3389/fgene.2026.1841048

**Published:** 2026-07-06

**Authors:** Sierra R. Sebesta, Emilie C. Baker, Kubra Z. Cilkiz, Rodolfo C. Cardoso, Thomas B. Hairgrove, Charles R. Long, Ronald D. Randel, Thomas H. Welsh, David G. Riley

**Affiliations:** 1 Department of Animal Science, College Station, Texas A&M University, College Station, TX, United States; 2 Texas A&M AgriLife Research and Extension Center, Overton, TX, United States

**Keywords:** *BEGAIN*, *HSPA6*, *TAT*, stress, sex differences

## Abstract

The liver is a central regulator of metabolic and endocrine functions that support fetal growth and postnatal development. Prenatal stress can reprogram hepatic development in the offspring, potentially causing long-term changes in metabolism and production efficiency. However, the influence of prenatal stress on hepatic molecular function and resulting phenotypes in beef cattle remains poorly understood. Therefore, the objectives of this study were to evaluate phenotypic traits and liver tissue gene expression in Brahman heifer and bull calves from prenatal transportation stress (PNS) and control (Control) treatment groups. One group of pregnant Brahman cows were transported for a 2-h period every 20 (±5) d from 60 to 140 days of gestation. Another group of pregnant Brahman cows served as Control. Thirty-two calves, eight heifer and eight bull calves from the PNS and Control groups, respectively, were utilized. Calves were weighed at approximately 25 (±2) d of age. The following day, calves were euthanized, and liver tissues were harvested. Phenotypic traits evaluated include birth weight, harvest weight, liver weight, pen score, and liver weight:harvest weight. Interaction of sex and treatment did not explain substantial variation for any trait (*P* > 0.28). Sex influenced birth weight, harvest weight, liver weight (*P* < 0.05), and treatment influenced liver weight:harvest weight (*P* < 0.10). Linear regression coefficients of traits on calf age as a linear covariate were not different from 0 (*P* > 0.41). Controlling false discovery rate at 0.10, there were no differentially expressed genes for PNS relative to Control and 13 differentially expressed genes for male relative to female comparisons. No genes were found to be differentially expressed across all comparisons. It is possible that the few differences between sexes and treatments may be due to relatively small sample sizes or to an unknown adaptation mechanism to the stress prenatally induced by this young age. Heightening the severity of prenatal transportation stress through increased duration or frequency of transportation may result in more differentially expressed genes.

## Introduction

The liver holds a vital position in various organ systems (Kalra et al., 2023). It is involved in several physiological processes and plays an essential role in many functions that are required for daily life (Pinsent, 1983). Filtration, storage, metabolism, excretion, immune defense, and hormone production all take place in the liver during times of eustress and especially may be perturbed under a variety of stress situations (Janovick-Guretzky et al., 2007). Stress is a response that takes place when an animal recognizes a threat to homeostasis, or a stable internal environment (Moberg, 2000). Stress can elicit negative implications on the liver (Swain, 2000). Transportation of cattle can induce both physical and psychological stress to differentially affect individual animals. Transportation of gestating Brahman cows has been confirmed to alter cortisol profiles and multiple hypothalamic-pituitary-adrenal axis functions of calves and the cows themselves (Lay et al., 1997; Price et al., 2015; Littlejohn et al., 2016). The presence of stressors during gestation can have impacts on the physiological functions of a bovine fetus. Elevated levels of maternal cortisol due to transportation stress during gestation may have effects on the growth and development of the offspring (Lay et al., 1997). Prenatal transportation stress may also influence the genes that are expressed in the liver of the calves.

Gene expression analysis may offer insight on transcriptional behavior. It provides quantitative details of RNA molecules within tissues across treatments or conditions (Lovén et al., 2012). Normal processes of the liver can be disrupted by physiological stressors. Due to the liver’s dynamic role in metabolism, vasculature, immunity, secretion and excretion, a change in physiological state caused by stress could impair liver function and alter gene expression (Janovick-Guretzky et al., 2007). Liver gene expression has been reported relative to bovine residual feed intake, rate of gain, and with various feed additives and nutritional restriction as treatments (McCabe et al., 2012; Tizioto et al., 2015; Zarek et al., 2017). These treatment conditions were imposed to calves postnatally when dairy and beef cattle attained various ages and stages of production. The impacts of prenatal transportation stress on gene expression have been evaluated in the amygdala of mature Brahman cows and Brahman calves (Baker et al., 2022; 2024), as well as stress axis tissues of mature Brahman cows (Earnhardt-San et al., 2025). Nutritional stress resulted in an enormous number of differentially expressed genes in the liver of dairy cows (McCabe et al., 2012), suggesting that this organ in bovine is especially stress sensitive. However, the influence of prenatal transportation stress on liver gene expression in young calves, in reasonable proximity to the stressor, has not been documented. The hypothesis was that liver gene expression would be altered by prenatal stress. The objectives of this research were to: 1) compare phenotypes expressed in Brahman heifer and bull calves exposed to prenatal transportation stress and a control, and 2) evaluate gene expression in liver tissues from Brahman heifer and bull calves within those groups.

## Materials and methods

All procedures followed the Guide for the Care and Use of Agricultural Animals in Research and Teaching (FASS, 2020) and were approved by the Texas A&M AgriLife Research Animal Use and Care Committee.

### Animal procedures

Mature Brahman cows, approximately 6 years of age, were utilized to evaluate differences in calf phenotype and liver gene expression. The Brahman cows were artificially inseminated with semen from a single Brahman bull in early May of 2018. Cows were assigned randomly to treatments with respect to temperament, age, and parity and were managed under the same environmental and nutrient conditions at the Texas A&M AgriLife Research and Extension Center at Overton. These include *ad libitum* coastal bermudagrass pastures (*Cynodon dactylon*) in the summer and fall, rye (*Secale cereale*) and ryegrass (*Lolium multiflorum*) overseeded pasture, and supplementation of coastal bermudagrass hay and 3:1 corn/soybean meal mix as needed. One group of pregnant Brahman cows were transported for a 2-h period every 20 days (±5 days) from 60 to 140 days of gestation as previously reported (Lay et al., 1997; Price et al., 2015; Littlejohn et al., 2016). A 3-section trailer (2.4 b y 7.3 m) towed by a three-quarter ton truck was utilized to transport the cows. The same individual drove on smooth paved highways each time for a total of 2-h at an average speed of 75 km per hour. This prenatal transportation treatment has been previously reported to increase maternal circulating concentrations of cortisol, a key hormone used as a stress marker (Price et al., 2015). The second group of pregnant Brahman cows was preserved as a non-transported control. A total of 32 calves, eight heifer and eight bull calves from the transported (PNS) and control (Control) groups, respectively, were utilized. At approximately 25 days (±2 days) of age, the calves were weighed, and pen temperament scores were collected. A single experienced evaluator individually assigned pen temperament scores to calves in groups of 3 to 5 animals within a confined pen. Calf reactions to the evaluator were assessed on a scale of 1 to 5 (Hammond et al., 1996). A pen temperament score of one was defined as nonaggressive or docile, and five was defined as easily excitable, very aggressive, or dangerous. One day after weighing and collection of pen scores, the calves were humanely euthanized using jugular intravenous administration 1 mL/4 kg live weight of barbiturate overdose (1 mL = 390 mg pentobarbitol and 50 mg phenytoin; Beuthanasia-D Solution, Merck Animal Health, Whitehouse Station, NJ, United States), and tissues, including the liver, were harvested. Livers from each calf were weighed and then sections of parenchyma of the right lobe, approximately 2–3 cm in length and 0.1–3 cm in thickness, were dissected aseptically for histology. Samples were frozen in liquid nitrogen and kept at −80 °C until assays were performed. Calf birth weights, harvest weights, liver weights, pen scores, and liver weight as a proportion of harvest weight were recorded.

### Preparation and sequencing

Liver tissue samples were submitted to Novogene Corporation (Davis, CA) for RNA quantification, library preparation, and transcriptome sequencing for gene expression analyses. RNA sample preparation input material consisted of 1 μg RNA per sample. NEBNext UltraTM RNA Library Prep Kit for Illumina (New England BioLabs, Ipswich, MA, United States) was utilized to generate sequencing libraries. Purification of mRNA was performed using poly-T oligo-attached magnetic beads. Fragmentation was carried out, first and second strand cDNA were synthesized, and remaining overhangs were converted into blunt ends. Ligation was conducted using NEBNext Adaptors after adenylation of 3′ ends of DNA fragments. Library fragments were purified with the AMPure XP system (Beckman Coulter, Brea, CA, United States) to select for cDNA fragments of 150–200 bp in length. Three μl of USER Enzyme (New England BioLabs) were used with size-selected, adapter-ligated cDNA at 37 °C for 15 min. The process was repeated for 5 min at 95 °C before polymerase chain reaction (PCR) was performed. Purification of the PCR products was performed (AMPure XP system) and quality of the library was evaluated using the Bioanalyzer 2,100 system (Agilent Technologies, Santa Clara, CA, United States). Index-coded samples were clustered, followed by sequencing of library preparations on an Illumina 1.9 platform (Illumina, San Diego, CA) and generation of paired-end reads.

RNA-seq libraries generated an average of 24 million reads per sample. Raw RNA FASTQ files were evaluated using FastQC (Babraham Bioinformatics, Cambridge, UK) and MultiQC (Babraham Bioinformatics) before undergoing quality control processing using TrimGalore (Babraham Bioinformatics) to remove adapters and low-quality reads. A read quality cutoff of 20 was utilized, which is the default based on phred-33 encoding (Illumina 1.9). A minimum length of 36 base pairs was required. After trimming was complete, reads were reevaluated with FastQC and MultiQC to ensure that they contained high quality, clean data for further analyses. Sequence duplication levels were approximately 70% across 32 bovine liver samples, which is consistent with the transcriptionally specialized nature of liver tissues. The average quality score after trimming was 35 and no samples had adapter contamination. All reads mapped successfully and none were duplicated.

The Spliced Transcripts Alignment to a Reference (STAR; Dobin et al., 2013) program was used to create a genome index file using the *Bos taurus* ARS-UCD1.2 genome assembly (Rosen et al., 2020). The clean, paired-end reads were then aligned to that index file and binary alignment map (BAM) files were generated for each sample. Read totals at each gene were then quantified from the BAM files using HTSeq (Anders et al., 2015). Gene level quantification using HTSeq helped reduce the impact of duplicate reads on downstream differential gene expression analysis, and identified approximately 13,000 expressed genes per sample, based on non-zero read counts.

### Statistical analyses

Statistical analyses of traits were performed using the lm function in R software (The R Foundation for Statistical Computing, Vienna, AT). Traits included calf birth weight, harvest weight, liver weight, pen score, and liver weight:harvest weight. Fixed classification effects investigated included sex and treatment main and interaction effects. Calf age in days was investigated as a linear covariate in all analyses except birth weight. Effects were considered significant when the *F* ratio of the effect had probability <0.05. Effects with *F* ratios that had probability between 0.05 and 0.1 were considered to be trends.

Read counts for each gene were evaluated using EdgeR (Robinson et al., 2010) to statistically evaluate differences in liver gene expression.

Comparisons included:

1. PNS males relative to Control males
2. PNS females relative to Control females
3. PNS males relative to PNS females
4. Control males relative to Control females
5. Males relative to females (treatments combined)
6. PNS calves relative to Control calves

**FIGURE 1:**
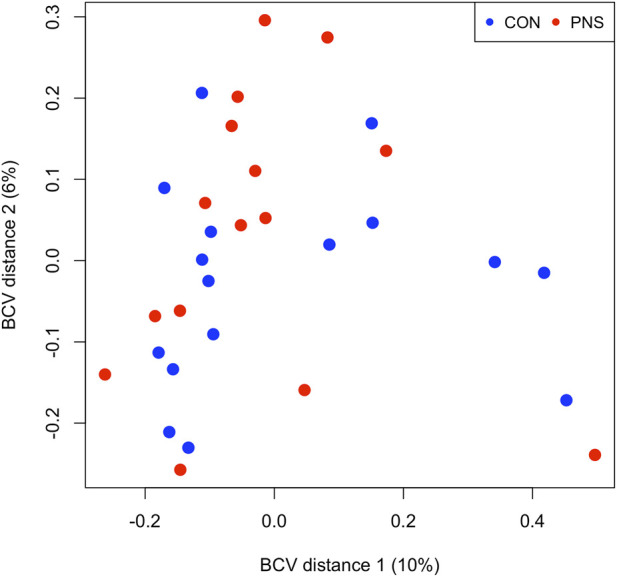
Multidimensional scaling plot with normalized values for prenatally stressed relative to control samples after outlier removal; BCV: biological coefficient of variation method.

**FIGURE 2:**
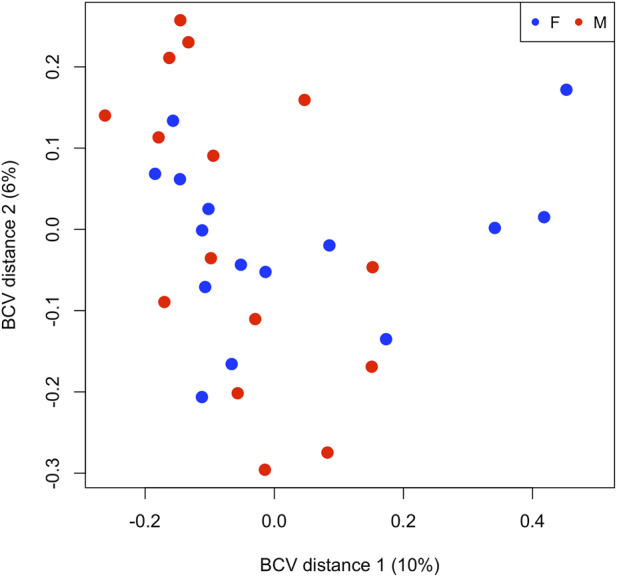
Multidimensional scaling plot with normalized values for male relative to female samples after outlier removal; BCV: biological coefficient of variation method.

Multidimensional scaling (MDS) plots were generated to visualize levels of similarity for each comparison prior to filtering genes with low expression. Genes with counts per million (CPM) values above three in at least five of the libraries were kept and those with low expression were removed. Subsequently, MDS plots with filtered values were created utilizing the biological coefficient of variation (BCV) method. An outlier sample was visually detected based on the MDS plots (Supplementary Figures 1, 2) and confirmed as > 0.3 BCV from the average BCV of the remaining samples (BCV distance 2), which led to its exclusion (Figures 1, 2). Normalization factors were calculated from raw gene-level counts using the edgeR’s (v4.8.0) trimmed mean of M-values (TMM) method, which scales each library to account for differences in sequencing depth and RNA composition by down-weighting genes with very large counts or extreme fold changes (Robinson et al., 2010). These TMM-derived scaling factors were used to adjust library sizes, and TMM-normalized counts were converted to CPM for downstream analyses. A generalized linear model (GLM) method was used to analyze the data. A negative binomial model was fitted to the Cox-Reid transformed dispersion estimates and the GLM function was fitted to the read counts and dispersion tables to use the GLM likelihood ratio test for each comparison (PNS minus control; male minus female). Each comparison was evaluated as a single factorial model. The 15 most variable genes were identified in each comparison, and heatmaps were created for each comparison. Differential expression was defined at false discovery rate (FDR) < 0.10 (Benjamini and Hochberg, 1995). Genes that were differentially expressed were submitted to the PANTHER Classification System v19.0 (Thomas et al., 2003) for annotation and functional enrichment analysis, using an FDR threshold of 0.05 to define significant categories.

## Results

### Phenotypic analyses

Main effect means and SE were calculated for sex and treatment for each trait and estimated linear regression coefficients and SE for calf age in days are presented in Table 1. The interaction of sex and treatment was not influential on any trait (*P* > 0.28). Sex was influential in analyses of calf birth weight, harvest weight, and liver weight (*P* < 0.05). Treatment was determined to be influential for calf liver weight:harvest weight (*P* < 0.10). As expected, males were heavier (*P* < 0.05) than females at birth and harvest and had heavier liver weights. No sex differences were observed (*P* > 0.25) for pen score or liver weight:harvest weight. Prenatally stressed (PNS) calves had heavier liver weight:harvest weights (*P* < 0.10) than Control calves. No treatment differences were found for the remaining four traits (*P* > 0.16). Age was not influential on harvest weight, liver weight, temperament score, or liver weight:harvest weight (*P* > 0.41).

**TABLE 1:** Means and estimates ±SE for traits (n = 32)^a^.

Effect	Level	Birth weight (kg)	Harvest weight (kg)	Liver weight (g)	Temperament score^b^	Liver weight: Harvest weight (g/kg)
Sex^c^	Female Male *P* Value	36.1 ± 1.29 40.5 ± 1.29 0.02	55.8 ± 2.11 63.0 ± 2.11 0.03	1,282 ± 55.4 1,475 ± 55.4 0.02	3.5 ± 0.26 3.1 ± 0.26 0.26	23.0 ± 0.51 23.5 ± 0.51 0.62
Treatment^c^	Control PNS *P* Value	39.6 ± 1.29 37.0 ± 1.29 0.17	60.0 ± 2.10 58.7 ± 2.10 0.68	1,360 ± 55.0 1,398 ± 55.0 0.64	3.3 ± 0.26 3.4 ± 0.26 0.80	22.6 ± 0.51 23.9 ± 0.51 0.09
Age^d^	- *P* Value	- -	0.3 ± 0.94 0.42	−8.2 ± 24.70 0.81	0.1 ± 0.12 0.70	−0.2 ± 0.23 0.53

a Effects were considered statistically significant when *P* < 0.05.

b Pen temperament scores for each calf were determined by willingness to be approached by a human and range from 1 (very calm) to five (easily excitable).

c The interaction of sex and treatment was not influential on any trait (*P* > 0.28).

d Estimated linear regression coefficients of traits on calf age in days ±SE.

### Differential gene expression

**FIGURE 3:**
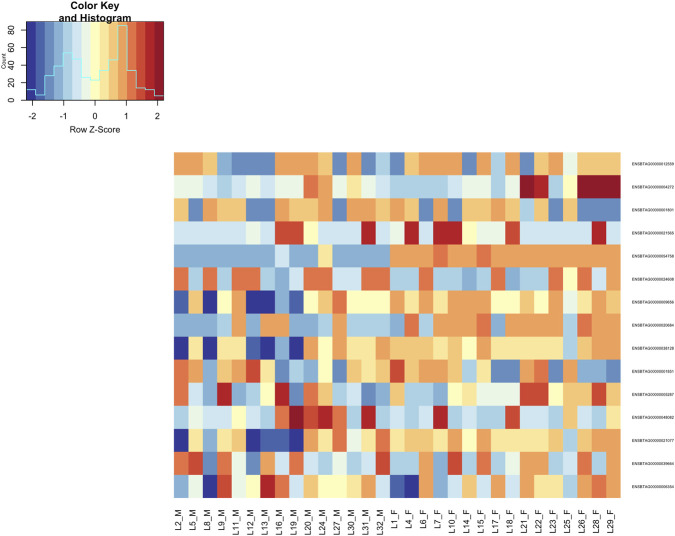
Heatmap representing the 15 most variable genes across samples for prenatally stressed relative to control samples. The Z score is a statistical measurement of a value in comparison to the group mean. The intensity of either the red or blue coloration at each intersection is indicative of the level of expression above or below the group mean for the specific gene ID in each sample.

PNS vs. Control. No genes were determined to be differentially expressed in PNS calves relative to Control calves with FDR controlled at 0.10. A heatmap of the 15 most variable genes across samples for PNS relative to Control samples is depicted as Figure 3. Gene ID and sample interactions high above or below the mean were minimal. In the two-way comparison of male PNS and male control calves, a total of 11 genes were found to be differentially expressed (FDR< 0.08; Table 2). Of these, eight genes were upregulated, and three were downregulated in male PNS compared to male control calves. The two-way comparison of female PNS and female control calves revealed no differentially expressed genes (FDR <0.10).

**TABLE 2:** Differentially expressed genes in male prenatally stressed calves relative to control male calves.

Gene ID	Gene name	FDR	Log_2_FC^a^	*Bos taurus* chromosome (BTA:Mb)
ENSBTAG00000011647	Solute carrier family 25 (mitochondrial carrier_ ornithine transporter) member 15 (*SLC25A15)*	0.002	1.618	12:21.78
ENSBTAG00000051412	Hemoglobin subunit alpha (*HBA)*	0.017	2.094	25:21.64
ENSBTAG00000005998	CCR4 carbon catabolite repression 4-like (*S. cerevisiae*) (*NOCT)*	0.017	1.721	17:18.58
ENSBTAG00000010431	LOC616371 protein (*LURAP1L)*	0.017	1.265	8:31.50
ENSBTAG00000049496	Cytochrome P450 (*CYP2C281)*	0.023	2.737	26:16.55
ENSBTAG00000005244	RAS like family 11 member a (*RASL11A)*	0.023	−1.342	12:32.60
ENSBTAG00000038112	MHC class I-like antigen recognition-like domain-containing protein (*LOC100850276)*	0.023	−2.529	9:84.72
ENSBTAG00000002214	Tyrosine aminotransferase (*TAT)*	0.043	0.915	18:39.48
ENSBTAG00000037644	Hemoglobin subunit beta (*HBB)*	0.044	2.459	15:48.36
ENSBTAG00000039035	Heat shock protein family a (Hsp70) member 6 (*HSPA6)*	0.060	4.005	3:80.32
ENSBTAG00000001376	RAD21 cohesin complex component like 1 (*RAD21L1)*	0.070	−0.907	13:59.87

a Log_2_FC, Logarithmic Fold Change: positive (negative) fold change indicates that prenatally stressed males had increased (decreased) expression relative to control males.

**FIGURE 4:**
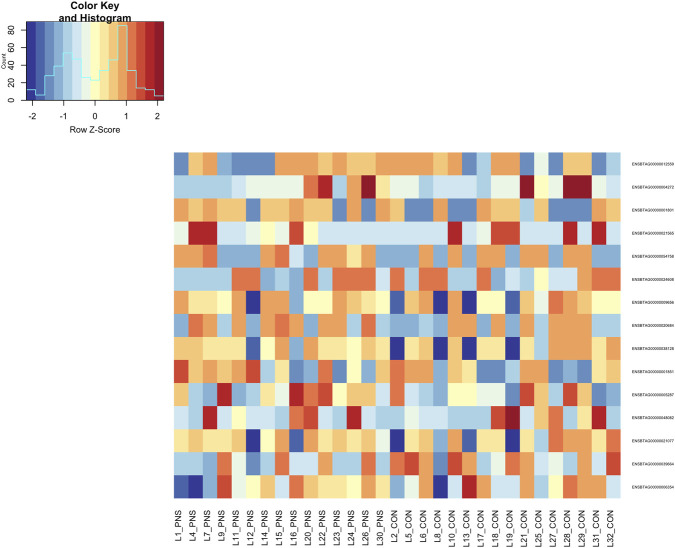
Heatmap representing the 15 most variable genes across samples for male relative to female samples. The Z score is a statistical measurement of a value in comparison to the group mean. The intensity of either the red or blue coloration at each intersection is indicative of the level of expression above or below the group mean for the specific gene ID in each sample.

*Male* vs. *Female.* With FDR controlled at 0.10, three genes were upregulated, and 10 genes were downregulated in male calves relative to female calves across treatments (Table 3). Visual portrayal of the 15 most variable genes across samples highlights a few detectable interactions, however none corresponded to the identified differentially expressed genes (Figure 4). Two-way analysis of PNS males and PNS females resulted in identification of three upregulated and four downregulated genes, respectively (Table 4). A total of 35 differentially expressed genes were identified in the two-way analysis of Control males and Control females (Table 5). Of these genes, seven were upregulated and 28 were downregulated in control males compared to control females.

**TABLE 3:** Differentially expressed genes in male calves relative to female calves across treatments.

Gene ID	Gene name	FDR	Log_2_FC^a^	*Bos taurus* chromosome (BTA:Mb)
ENSBTAG00000054758	Unknown	<0.001	−6.201	X:77.19
ENSBTAG00000024874	Mitotic arrest deficient 2 like 1 (*MAD2L1)*	<0.001	−1.358	Un_NW_020190807v1:7,006–14630
ENSBTAG00000014337	Eukaryotic translation initiation factor 2 subunit 3 (*EIF2S3)*	0.002	−0.440	X:11.92
ENSBTAG00000019616	Serum amyloid P-component (*APCS)*	0.003	−2.448	3:10.13
ENSBTAG00000003740	[Histone H3]-trimethyl-L-lysine (27) demethylase (*KDM6A)*	0.003	−0.427	X:98.64
ENSBTAG00000017716	Brain enriched guanylate kinase associated (*BEGAIN)*	0.011	−0.914	21:65.42
ENSBTAG00000053806	Bone marrow stromal antigen 2 (*BST2)*	0.019	−2.454	7:57.45
ENSBTAG00000000277	Interleukin-18 (*IL18)*	0.064	−0.665	15:22.48
ENSBTAG00000000207	Haloacid dehalogenase-like hydrolase domain containing 1 A (*PUDP)*	0.067	0.581	X:13.56
ENSBTAG00000007881	Interferon induced protein with tetratricopeptide repeats 1 (*IFIT1)*	0.070	−2.232	26:11.07
ENSBTAG00000006185	Kinetochore protein Spc24 (*SPC24)*	0.073	0.697	7:15.59
ENSBTAG00000008471	Interferon induced GTP-binding protein Mx2 (*MX2)*	0.073	−2.169	1:14.16
ENSBTAG00000044427	Unknown	0.083	2.848	10:26.78

a Log_2_FC, Logarithmic Fold Change: positive (negative) fold change indicates that males had increased (decreased) expression relative to females.

**TABLE 4:** Differentially expressed genes in prenatally stressed (PNS) male calves relative to female calves.

Gene ID	Gene name	FDR	Log_2_FC^a^	*Bos taurus* chromosome (BTA:Mb)
ENSBTAG00000017716	Brain enriched guanylate kinase associated (*BEGAIN)*	<0.001	−1.239	21:65.42
ENSBTAG00000054758	Unknown	<0.001	−5.189	X:77.19
ENSBTAG00000020406	Glypican 3 (*GPC3)*	0.006	1.485	X:17.37
ENSBTAG00000043553	Glutathione peroxidase 3 (*GPX3)*	0.011	3.065	7:62.28
ENSBTAG00000032617	F-box protein 44 (*FBXO44)*	0.082	−1.601	16:42.02
ENSBTAG00000009788	Uncharacterized protein (*ACOT2)*	0.091	1.457	10:85.02
ENSBTAG00000014698	Caspase recruitment domain family member 11 (*CARD11)*	0.091	−0.669	25:40.42

a Log_2_FC, Logarithmic Fold Change: positive (negative) fold change indicates that prenatally stressed males had increased (decreased) expression relative to prenatally stressed females.

**TABLE 5:** Differentially expressed genes in Control male calves relative to female calves.

Gene ID	Gene name	FDR	Log_2_FC^a^	*Bos taurus* chromosome (BTA:Mb)
ENSBTAG00000054758	​	<0.001	−11.226	X:77.19
ENSBTAG00000024874	Mitotic arrest deficient 2 like 1 (*MAD2L1)*	<0.001	−1.691	Un_NW_020190807v1:6,359–14611
ENSBTAG00000007881	Interferon induced protein with tetratricopeptide repeats 1 (*IFIT1)*	0.001	−3.284	26:11.07
ENSBTAG00000043250	​	0.001	4.041	23:25.22
ENSBTAG00000044427	​	0.001	3.768	10:26.78
ENSBTAG00000019616	Serum amyloid P-component (*APCS)*	0.001	−3.123	3:10.13
ENSBTAG00000049916	​	0.002	3.430	10:42.63
ENSBTAG00000053806	Bone marrow stromal antigen 2 (*BST2)*	0.003	−3.166	7:57.46
ENSBTAG00000008471	Interferon induced GTP-binding protein Mx2 (*MX2)*	0.004	−2.927	1:14.16
ENSBTAG00000054661	​	0.004	3.063	2:12.16
ENSBTAG00000014707	Ubiquitin-like protein ISG15 (*ISG15)*	0.008	−2.700	16:51.46
ENSBTAG00000030913	Interferon-induced GTP-binding protein Mx1 (*MX1)*	0.008	−2.287	1:14.17
ENSBTAG00000006694	C-X-C motif chemokine ligand 14 (*CXCL14)*	0.010	1.678	7:47.07
ENSBTAG00000019979	Cytidine/Uridine monophosphate kinase 2 (*CMPK2)*	0.014	−2.157	11:90.07
ENSBTAG00000020536	HECT and RLD domain containing E3 ubiquitin protein ligase family member 6 (*HERC6)*	0.021	−2.437	6:36.31
ENSBTAG00000016061	Radical S-adenosyl methionine domain-containing protein 2 (*RSAD2)*	0.034	−2.451	11:90.04
ENSBTAG00000008021	Uncharacterized protein (*LOC112441507)*	0.035	−2.271	7:57.35
ENSBTAG00000016661	Ubiquitin specific peptidase 18 (*USP18)*	0.036	−1.885	5:76.00
ENSBTAG00000045588	Uncharacterized protein (*LOC100298356)*	0.037	−2.254	7:57.26
ENSBTAG00000052369	​	0.042	−1.085	Un_NW_020192089v1:77,605–79758
ENSBTAG00000053807	2′-5′ oligoadenylate synthase (*OAS1X)*	0.048	−2.835	17:61.38
ENSBTAG00000030932	Interferon induced protein 44 like (*IFI44L)*	0.048	−1.793	3:66.05
ENSBTAG00000034349	Interferon induced protein 44 (*IFI44)*	0.048	−1.950	3:66.02
ENSBTAG00000039861	2′-5′ oligoadenylate synthase (*OAS1Y)*	0.054	−2.136	17:61.36
ENSBTAG00000009768	Interferon-induced protein with tetratricopeptide repeats 3 (*IFIT3)*	0.063	−1.538	26:11.05
ENSBTAG00000050459	Family with sequence similarity 222 member a (*FAM222A)*	0.063	0.849	17:63.46
ENSBTAG00000003366	RNA helicase (*DDX58)*	0.064	−1.565	8:11.56
ENSBTAG00000001292	Lactotransferrin (*LTF)*	0.072	2.155	22:52.95
ENSBTAG00000052306	Interferon induced transmembrane protein 3 (*LOC777594)*	0.073	−1.360	29:50.93
ENSBTAG00000012335	Ubiquitin like modifier activating enzyme 7 (*UBA7)*	0.086	−1.774	22:50.42
ENSBTAG00000008909	Polyribonucleotide nucleotidyltransferase (*PNPT1)*	0.095	−1.199	11:38.31
ENSBTAG00000012406	Z-DNA binding protein 1 (*ZBP1)*	0.095	−1.383	13:58.56
ENSBTAG00000020166	Zinc finger NFX1-type containing 1 (*ZNFX1)*	0.095	−1.593	13:77.35
ENSBTAG00000046580	RNA helicase (*DHX58)*	0.095	−1.417	19:42.24
ENSBTAG00000009933	E3 ubiquitin-protein ligase (*DTX3L)*	0.095	−1.494	1:66.99

a Log_2_FC, Logarithmic Fold Change: positive (negative) fold change indicates that control males had increased (decreased) expression relative to control females.

### Gene ontology

PNS vs. Control. A single molecular function was significantly enriched (FDR < 0.05): “tetrapyrrole binding”. Differentially expressed genes were also enriched in the cellular component terms “haptoglobin–hemoglobin complex” and “hemoglobin complex” (Table 6). Pathways and functions of differentially expressed genes were not evaluated in PNS relative to control calves, both across sexes and within females, because no genes identified as differentially expressed in those comparisons. (FDR < 0.10).

**TABLE 6:** Significant (FDR < 0.05) Gene ontology molecular function and cellular component terms for genes differentially expressed between male prenatally stressed and male control calves.

Term	Ontology	FDR	Genes
Tetrapyrrole binding (GO:0046906)	Molecular function	0.0466	ENSBTAG00000037644; ENSBTAG00000051412; ENSBTAG00000049496
Haptoglobin–hemoglobin complex (GO:0031838)	Cellular component	0.0191	ENSBTAG00000037644; ENSBTAG00000051412
Hemoglobin complex (GO:0005833)	Cellular component	0.0136	ENSBTAG00000037644; ENSBTAG00000051412

**FIGURE 5:**
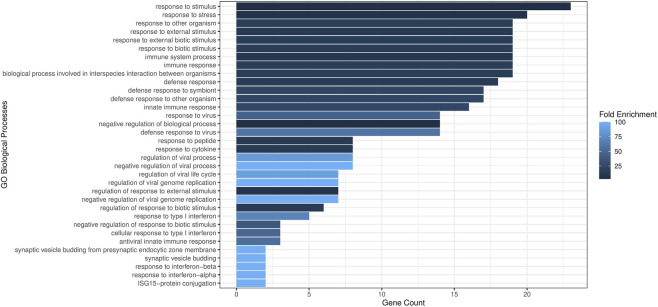
Significant (FDR < 0.05) Gene ontology (GO) biological processes from genes differentially expressed between control male and control females.

Male vs. Female. No gene ontology categories reached significance (FDR < 0.05) for genes differentially expressed between males and females or for genes differentially expressed between PNS males and females. Within the Control male vs. Control female comparison, numerous (Figure 5) biological processes identified as significant, with many genes involved in processes relating to the immune system including “immune response”, “response to cytokine” and “defense response to virus” (Supplementary Table 1). No molecular function, cellular component, or pathway terms were identified as significant (FDR <0.05) using these genes.

## Discussion

### Phenotypic analyses

The interaction of calf sex and treatment was not detected in analyses of calf birth weight, harvest weight, liver weight, pen score, or liver weight:harvest weight. Calf sex was influential in analyses of birth weight, harvest weight, and liver weight. As expected, male calves were heavier than female calves for these weight traits, which agrees with reported sex differences in birth weight for calves subjected to the same prenatal stress (Littlejohn et al., 2016). However, birth weights, harvest weights, and liver weights did not differ (P > 0.16) between the PNS and Control groups. This corresponds to results from McCabe et al. (2012) and Pegolo et al. (2016), in which liver weights and cattle growth traits, respectively, were not found to be different between nutritional treatment groups. Physiological changes related to fat were found between cattle consuming different diet treatments (Kim et al., 2022). In the present study, significant treatment differences were observed only for calf liver weight:harvest weight. PNS calves had larger liver weights and smaller harvest weights compared to Control calves, but neither of these differences were significant (P > 0.63). Littlejohn et al. (2016) also reported that prenatal transportation stress had no effect on calf birth weight. Neither sex nor treatment explained significant variation in calf pen temperament scores. This contradicts almost all reports that female cattle were more temperamental than males (e.g., Shrode and Hammack, 1971; Hoppe et al., 2010; Littlejohn et al., 2016). Consistent with Littlejohn et al. (2016), PNS calves had worse temperament than Control calves. The present study is unique from others in that calves were not weaned, that is, they were nursing dams.

### Differential gene expression and gene ontology

Differential expression of genes between animals is an appropriate method to identify candidate genes and target certain traits (Tizioto et al., 2015). Gene expression analysis of liver tissues indicated few differentially expressed genes by prenatal stress category. This aligns with prior research in which few differences in gene expression were identified in amygdala between of PNS and Control 5-year-old Brahman cows and 25-day-old Brahman calves (Baker et al., 2022; Baker et al., 2024). One gene that had increased expression in prenatally stressed male calves was tyrosine aminotransferase (*TAT*), a glucocorticoid-responsive enzyme. Prior work has shown that glucocorticoid receptor activation can induce stable epigenetic changes at the TAT promoter in liver (Krontira et al., 2020), suggesting a potential pathway by which prenatal stress-related glucocorticoid exposure could contribute to altered hepatic gene expression in offspring. The gene that showed the largest difference in expression between prenatally stressed and control males (log_2_FC = 4.005) was Heat shock protein family A (Hsp70) member 6 (*HSPA6*), an inducible Hsp70-family chaperone. Higher hepatic Hsp70 protein levels have been reported in neonatal intrauterine growth-restricted piglets compared with normal-birth-weight piglets (Li et al., 2012).

The timing and level of severity of stress may have influence on the extent of gene expression alterations. Offspring of mothers exposed to an acute natural disaster at different stages of gestation showed stage-dependent differences in placental expression of stress-related genes (Zhang et al., 2018), and more severe maternal stress has been linked to more pronounced alterations in offspring stress-related gene regulation (Palma-Gudiel et al., 2015). Given the dynamic nature of liver development across gestation (Mao et al., 2008), the timing of prenatal stress is also likely to influence the extent to which it can alter hepatic growth and gene expression. Therefore, the level of prenatal transportation stress utilized may not be considerable enough to result in significant effects.

Controlling FDR at 0.10, more genes were differentially expressed between sexes than between treatment groups. Sex differences in liver transcriptome profiles have been observed previously in dairy calves (Thompson et al., 2025). Sex differences in hepatic gene expression are well documented in mammals, particularly in rodent models, where they are strongly influenced by growth hormone signaling (Waxman and O’Connor, 2006). Five genes were differentially expressed between male and female calves, as well as control male and control female calves. These include mitotic arrest deficient two like 1 (*MAD2L1*), serum amyloid P-component (*APCS*), bone marrow stromal antigen 2 (*BST2*), interferon induced protein with tetratricopeptide repeats 1 (*IFIT1*), and interferon induced GTP-binding protein Mx2 (*MX2*). These genes are related to checkpoint signaling (Sadkowski et al., 2006), innate immune response (Niribili et al., 2024), pain (Katano et al., 2023), and defense response to virus (Takeda et al., 2012; Tirumurugaan et al., 2020). Additionally, these genes were annotated to various cellular components such as “cytoplasm”, “extracellular space”, “synapse”, “Golgi apparatus”, “cell surface”, “cytosol” and “nucleus”. These biological processes and cellular components align with those found in research by Serna-García et al. (2023) and Mukiibi et al. (2019). One gene, brain enriched guanylate kinase associated (*BEGAIN*), was differentially expressed in both the male and female calf comparison, and the PNS male and PNS female comparison. Brain enriched guanylate kinase associated encodes a brain-enriched synaptic protein implicated in learning, memory, and neuropathic pain signaling (Wei et al., 2023); however, its involvement in prenatal stress responses or liver function has not, to our knowledge, been described.

The liver has a central role in innate immunity, where hepatocytes and innate immune cells mediate pattern-recognition and type I interferon–driven antiviral defense (Xu et al., 2021). Functional enrichment analysis of genes that were differentially expressed between control males and females indicated a strong enrichment of innate immune and antiviral defense processes, particularly type I interferon signaling and pathways involved in viral response and host defense. In humans, liver innate and type I interferon–mediated antiviral responses have been linked to sex specific differences in the course and outcome of chronic HBV and HCV infection, reflecting the impact of sex hormones on antiviral and inflammatory signaling in this tissue (Ruggieri et al., 2018). These sex differences in hepatic immunity exist even in the absence of infection (Burra et al., 2024). Genes involved with immune function were also identified in prior bovine gene expression research (Zarek et al., 2017; Sosa et al., 2022).

Several genes that had increased expression in females relative to males are located on the X chromosome. While dosage compensation through X inactivation typically equalizes X-linked gene expression between sexes, some X-linked genes escape this process and therefore can show higher mRNA expression in females. The histone demethylase gene Utx (*UTX*) is one gene that escapes X inactivation in both mice and humans (Greenfield et al., 1998). The translation-initiation factor subunit gene (*EIF2S3)* likewise escapes X inactivation and has been reported to exhibit female-biased mRNA expression in adult liver (Xu et al., 2006). Our findings are consistent with incomplete dosage compensation of X-escapee genes, as reflected by the higher hepatic expression of *UTX* and *EIF2S3* in females.

Other bovine gene expression studies have reported differentially expressed genes between different sexes or nutritional treatments; however, those genes did not align with those identified in this research (Seo et al., 2016; Coelho et al., 2022). This may be due to liver transcriptomes of different cattle breeds having divergent genetic profiles (Pareek et al., 2019). No genes were differentially expressed across all comparisons in the study.

Results must be considered with respect to age of calves. Age-dependent changes in gene expression must be likely in many tissues (e.g., Chang et al., 2023; Zeller et al., 2025). This has been characterized in bovine associated with sexual maturation (Wanjiru et al., 2025) but is not documented in liver. Profound changes associated with diet and weaning undoubtedly alter the gene expression profiles in some ways. The calves sampled likely had almost entirely milk as their primary source of nutrients; milk as compared to other later diets has not been characterized with respect to gene expression and certainly would have age confounding. The sample size may be responsible for the limited numbers of differentially expressed genes in the different comparisons.

## Conclusion

No interaction of sex with prenatal stress treatment was evident in analyses of birth weight, harvest weight, liver weight, pen score, or liver weight harvest weight in 25-day-old Brahman calves. Not surprisingly, bull calves had heavier birth, harvest, and liver weights. Calves exposed to prenatal transportation stress resulted in heavier liver weight as a proportion of harvest weight relative to controls. The minor differences in age were not influential in any phenotypic assessments. There were few differentially expressed genes between sexes and treatments. A partial natural offsetting of the negative impacts of prenatal stress may be possible by this young age in calves. Heightening the severity of prenatal transportation stress through increased duration or frequency of transportation may result in more differentially expressed genes. Alteration of prenatal transportation stress severity, time of year for transportation, animal age at harvest, and tissue sample collection area may be required to better understand the influence of prenatal transportation stress on phenotypic and genetic expression in beef cattle.

## Data Availability

The original contributions presented in the study are publicly available. This data can be found in the NCBI Sequence Read Archive (SRA) repository with the BioProject accession number PRJNA1478047 (SRA Study: SRP709295).
