# Supplementary figures and images for: Effects of prenatal transportation stress on liver gene expression in male and female Brahman calves
Source: Front Genet. 2026 Jul 6;17:1841048. doi: 10.3389/fgene.2026.1841048 (PMC13381019; doi:10.3389/fgene.2026.1841048)

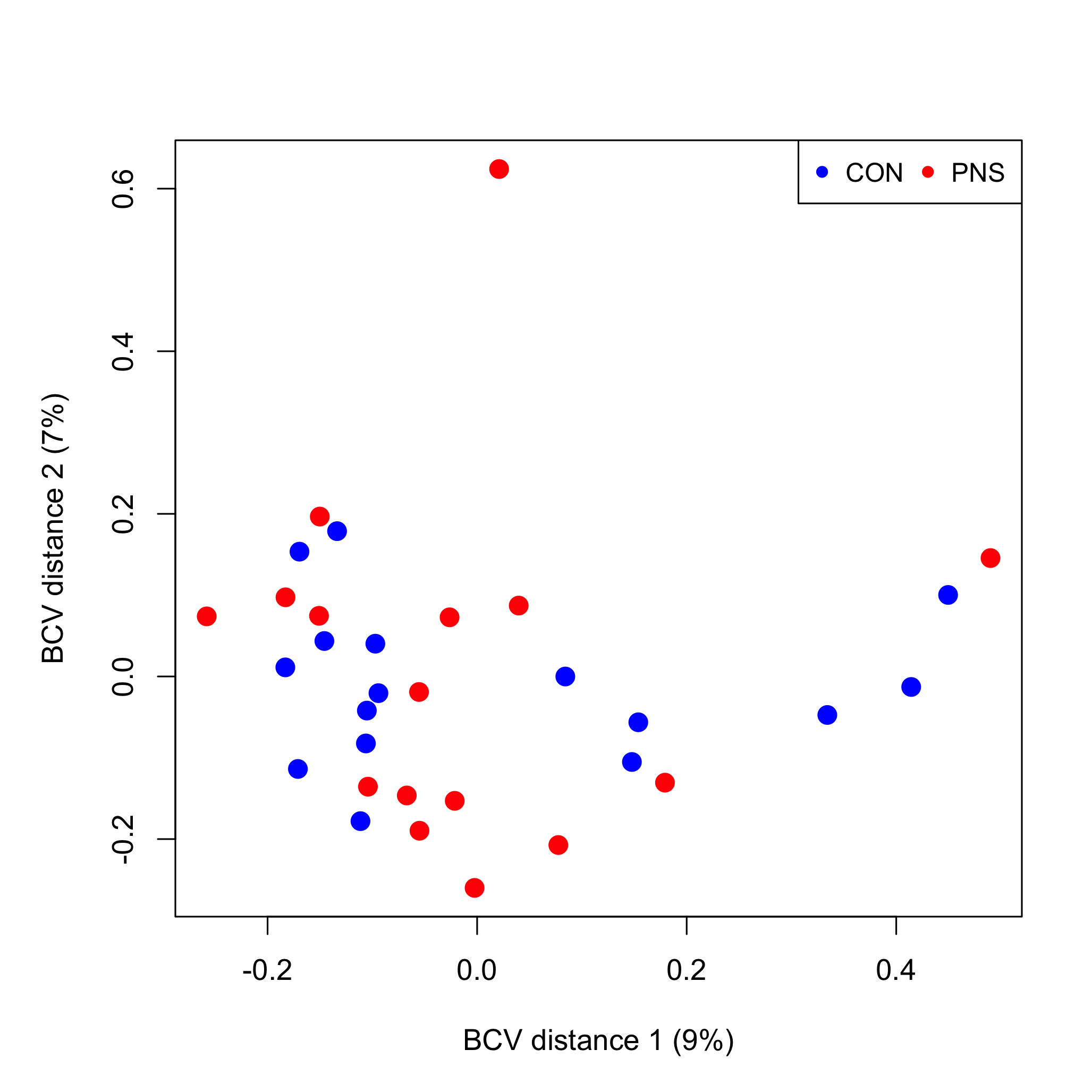

Supplement: Supplementary file 1 [file Image1.tiff]

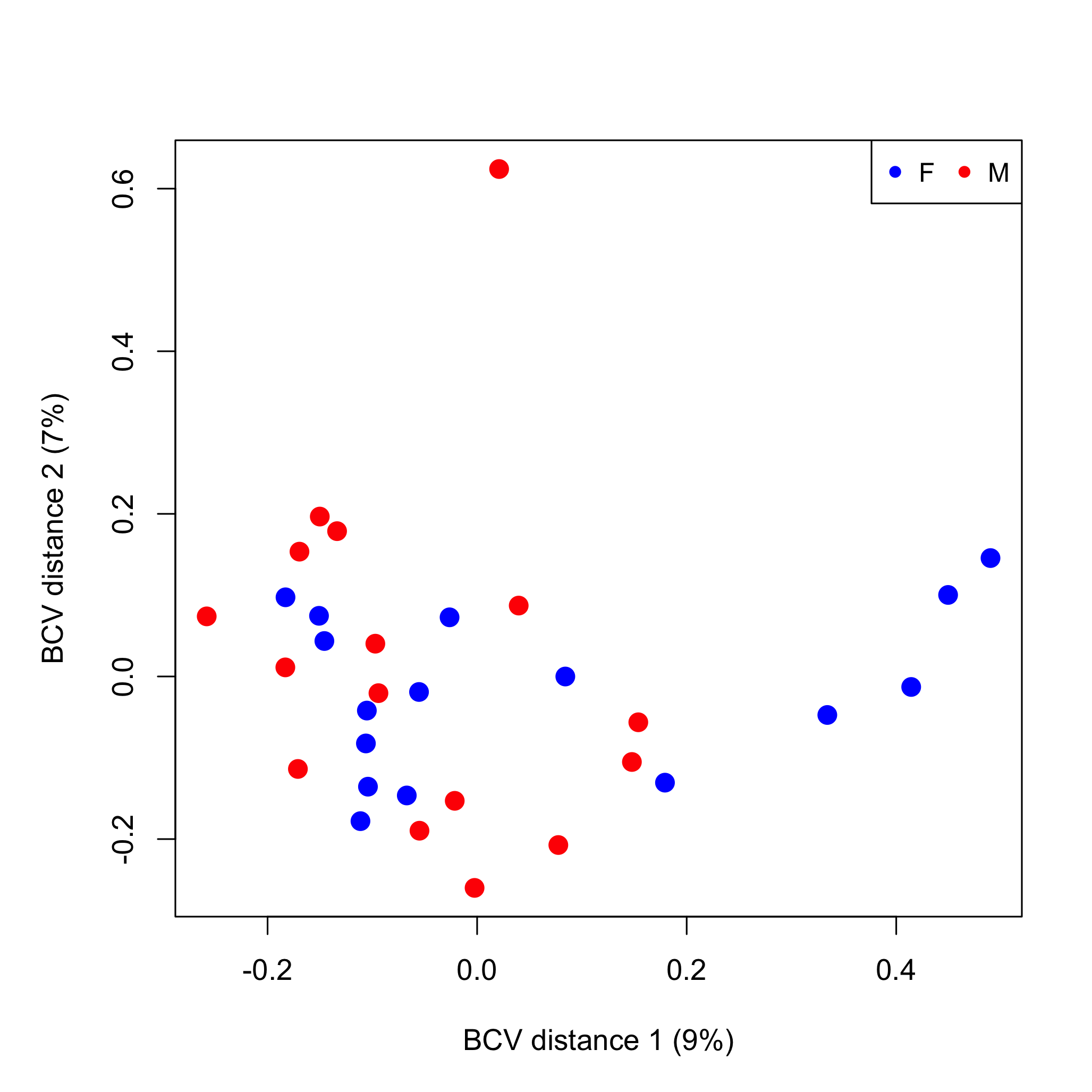

Supplement: Supplementary file 3 [file Image2.tiff]
