# Supplementary material for: Effects of prenatal transportation stress on liver gene expression in male and female Brahman calves
Source: Front Genet. 2026 Jul 6;17:1841048. doi: 10.3389/fgene.2026.1841048 (PMC13381019; doi:10.3389/fgene.2026.1841048)
Supplement: Supplementary file 2 [file DataSheet1.pdf]

frontiers

Supplementary Table 1: Significant biological processes and corresponding genes (FDR < 0.05) for genes differentially expressed between control male and females.

Term	FDR	Genes
antiviral innate immune response (GO:0140374)	0.009	ENSBTAG00000009768;ENSBTAG00000039861;ENSBTAG00000007881
biological process involved in interspecies interaction between organisms (GO:0044419)	< 0.001	ENSBTAG00000008021;ENSBTAG00000008471;ENSBTAG00000012335;ENSBTAG00000019979;ENSBTAG00000019616;ENSBTAG00000009768;ENSBTAG00000001292;ENSBTAG00000014707;ENSBTAG00000006694;ENSBTAG00000007881;ENSBTAG00000003366;ENSBTAG00000020166;ENSBTAG00000030913;ENSBTAG00000052306;ENSBTAG00000053806;ENSBTAG00000046580;ENSBTAG00000030932;ENSBTAG00000016061;ENSBTAG00000039861
cellular response to type I interferon (GO:0071357)	0.013	ENSBTAG00000052306;ENSBTAG00000039861;ENSBTAG00000007881
defense response (GO:0006952)	< 0.001	ENSBTAG00000008021;ENSBTAG00000008471;ENSBTAG00000012335;ENSBTAG00000019616;ENSBTAG00000009768;ENSBTAG00000001292;ENSBTAG00000014707;ENSBTAG00000006694;ENSBTAG00000007881;ENSBTAG00000003366;ENSBTAG00000020166;ENSBTAG00000030913;ENSBTAG00000052306;ENSBTAG00000053806;ENSBTAG00000046580;ENSBTAG00000030932;ENSBTAG00000016061;ENSBTAG00000039861
defense response to other organism (GO:0098542)	< 0.001	ENSBTAG00000008021;ENSBTAG00000008471;ENSBTAG00000012335;ENSBTAG00000019616;ENSBTAG00000009768;ENSBTAG00000001292;ENSBTAG00000014707;ENSBTAG00000006694;ENSBTAG00000007881;ENSBTAG00000003366;ENSBTAG00000020166;ENSBTAG00000030913;ENSBTAG00000052306;ENSBTAG00000053806;ENSBTAG00000046580;ENSBTAG00000016061;ENSBTAG00000039861
defense response to symbiont (GO:0140546)	< 0.001	ENSBTAG00000008021;ENSBTAG00000008471;ENSBTAG00000012335;ENSBTAG00000019616;ENSBTAG00000009768;ENSBTAG00000001292;ENSBTAG00000014707;ENSBTAG00000006694;ENSBTAG00000007881;ENSBTAG00000003366;ENSBTAG00000020166;ENSBTAG00000030913;ENSBTAG0

		0000052306;ENSBTAG00000053806;ENSBTAG00000046580;ENSBTAG00000016061;ENSBTAG00000039861
defense response to virus (GO:0051607)	< 0.001	ENSBTAG00000008021;ENSBTAG00000008471;ENSBTAG00000009768;ENSBTAG00000014707;ENSBTAG00000007881;ENSBTAG00000003366;ENSBTAG00000020166;ENSBTAG00000030913;ENSBTAG00000052306;ENSBTAG00000053806;ENSBTAG00000046580;ENSBTAG00000030932;ENSBTAG00000016061;ENSBTAG00000039861
immune response (GO:0006955)	< 0.001	ENSBTAG00000008021;ENSBTAG00000008471;ENSBTAG00000012335;ENSBTAG00000019616;ENSBTAG00000009768;ENSBTAG00000001292;ENSBTAG00000014707;ENSBTAG00000006694;ENSBTAG00000007881;ENSBTAG00000003366;ENSBTAG00000020166;ENSBTAG00000030913;ENSBTAG00000052306;ENSBTAG00000053806;ENSBTAG00000046580;ENSBTAG00000030932;ENSBTAG00000016061;ENSBTAG00000034349;ENSBTAG00000039861
immune system process (GO:0002376)	< 0.001	ENSBTAG00000008021;ENSBTAG00000008471;ENSBTAG00000012335;ENSBTAG00000019616;ENSBTAG00000009768;ENSBTAG00000001292;ENSBTAG00000014707;ENSBTAG00000006694;ENSBTAG00000007881;ENSBTAG00000003366;ENSBTAG00000020166;ENSBTAG00000030913;ENSBTAG00000052306;ENSBTAG00000053806;ENSBTAG00000046580;ENSBTAG00000030932;ENSBTAG00000016061;ENSBTAG00000034349;ENSBTAG00000039861
innate immune response (GO:0045087)	< 0.001	ENSBTAG00000008021;ENSBTAG00000008471;ENSBTAG00000012335;ENSBTAG00000019616;ENSBTAG00000009768;ENSBTAG00000001292;ENSBTAG00000014707;ENSBTAG00000007881;ENSBTAG00000003366;ENSBTAG00000020166;ENSBTAG00000030913;ENSBTAG00000052306;ENSBTAG00000053806;ENSBTAG00000046580;ENSBTAG00000016061;ENSBTAG00000039861
ISG15-protein conjugation (GO:0032020)	0.007	ENSBTAG00000012335;ENSBTAG00000014707
negative regulation of biological process (GO:0048519)	0.009	ENSBTAG00000019616;ENSBTAG00000009933;ENSBTAG00000001292;ENSBTAG00000014707;ENSBTAG00000006694;ENSBTAG00000007881;ENSBTAG00000020166;ENSBTAG00000030913;ENSBTAG00000024874;ENSBTAG00000052306;ENSBTAG00000046580;ENSBTAG00000016061;ENSBTAG00000039861;ENSBTAG00000008909

negative regulation of response to biotic stimulus (GO:0002832)	0.027	ENSBTAG00000046580;ENSBTAG00000001292;ENSBTAG00000014707
negative regulation of viral genome replication (GO:0045071)	< 0.001	ENSBTAG00000030913;ENSBTAG00000052306;ENSBTAG00000016061;ENSBTAG00000014707;ENSBTAG00000039861;ENSBTAG00000007881;ENSBTAG00000020166
negative regulation of viral process (GO:0048525)	< 0.001	ENSBTAG00000030913;ENSBTAG00000052306;ENSBTAG00000019616;ENSBTAG00000016061;ENSBTAG00000014707;ENSBTAG00000039861;ENSBTAG00000007881;ENSBTAG00000020166
regulation of response to biotic stimulus (GO:0002831)	0.002	ENSBTAG00000046580;ENSBTAG00000016061;ENSBTAG00000009933;ENSBTAG00000001292;ENSBTAG00000014707;ENSBTAG00000020166
regulation of response to external stimulus (GO:0032101)	0.008	ENSBTAG00000046580;ENSBTAG00000016061;ENSBTAG00000009933;ENSBTAG00000001292;ENSBTAG00000014707;ENSBTAG00000006694;ENSBTAG00000020166
regulation of viral genome replication (GO:0045069)	< 0.001	ENSBTAG00000030913;ENSBTAG00000052306;ENSBTAG00000016061;ENSBTAG00000014707;ENSBTAG00000039861;ENSBTAG00000007881;ENSBTAG00000020166
regulation of viral life cycle (GO:1903900)	< 0.001	ENSBTAG00000030913;ENSBTAG00000052306;ENSBTAG00000016061;ENSBTAG00000014707;ENSBTAG00000039861;ENSBTAG00000007881;ENSBTAG00000020166
regulation of viral process (GO:0050792)	< 0.001	ENSBTAG00000030913;ENSBTAG00000052306;ENSBTAG00000019616;ENSBTAG00000016061;ENSBTAG00000014707;ENSBTAG00000039861;ENSBTAG00000007881;ENSBTAG00000020166
response to biotic stimulus (GO:0009607)	< 0.001	ENSBTAG00000008021;ENSBTAG00000008471;ENSBTAG00000012335;ENSBTAG00000019979;ENSBTAG00000019616;ENSBTAG00000009768;ENSBTAG00000001292;ENSBTAG00000014707;ENSBTAG00000006694;ENSBTAG00000007881;

		ENSBTAG00000003366;ENSBTAG00000020166;ENSBTAG00000030913;ENSBTAG00000052306;ENSBTAG00000053806;ENSBTAG00000046580;ENSBTAG00000030932;ENSBTAG00000016061;ENSBTAG00000039861
response to cytokine (GO:0034097)	< 0.001	ENSBTAG00000030913;ENSBTAG00000008471;ENSBTAG00000052306;ENSBTAG00000009768;ENSBTAG00000014707;ENSBTAG00000039861;ENSBTAG00000007881;ENSBTAG00000008909
response to external biotic stimulus (GO:0043207)	< 0.001	ENSBTAG00000008021;ENSBTAG00000008471;ENSBTAG00000012335;ENSBTAG00000019979;ENSBTAG00000019616;ENSBTAG00000009768;ENSBTAG00000001292;ENSBTAG00000014707;ENSBTAG00000006694;ENSBTAG00000007881;ENSBTAG00000003366;ENSBTAG00000020166;ENSBTAG00000030913;ENSBTAG00000052306;ENSBTAG00000053806;ENSBTAG00000046580;ENSBTAG00000030932;ENSBTAG00000016061;ENSBTAG00000039861
response to external stimulus (GO:0009605)	< 0.001	ENSBTAG00000008021;ENSBTAG00000008471;ENSBTAG00000012335;ENSBTAG00000019979;ENSBTAG00000019616;ENSBTAG00000009768;ENSBTAG00000001292;ENSBTAG00000014707;ENSBTAG00000006694;ENSBTAG00000007881;ENSBTAG00000003366;ENSBTAG00000020166;ENSBTAG00000030913;ENSBTAG00000052306;ENSBTAG00000053806;ENSBTAG00000046580;ENSBTAG00000030932;ENSBTAG00000016061;ENSBTAG00000039861
response to interferon-alpha (GO:0035455)	0.020	ENSBTAG00000008471;ENSBTAG00000052306
response to interferon-beta (GO:0035456)	0.048	ENSBTAG00000052306;ENSBTAG00000008909
response to other organism (GO:0051707)	< 0.001	ENSBTAG00000008021;ENSBTAG00000008471;ENSBTAG00000012335;ENSBTAG00000019979;ENSBTAG00000019616;ENSBTAG00000009768;ENSBTAG00000001292;ENSBTAG00000014707;ENSBTAG00000006694;ENSBTAG00000007881;ENSBTAG00000003366;ENSBTAG00000020166;ENSBTAG00000030913;ENSBTAG00000052306;ENSBTAG00000053806;ENSBTAG00000046580;ENSBTAG00000030932;ENSBTAG00000016061;ENSBTAG00000039861

response to peptide (GO:1901652)	< 0.001	ENSBTAG00000030913;ENSBTAG00000008471;ENSBTAG00000052306;ENSBTAG00000009768;ENSBTAG00000014707;ENSBTAG00000039861;ENSBTAG00000007881;ENSBTAG00000008909
response to stimulus (GO:0050896)	< 0.001	ENSBTAG00000008021;ENSBTAG00000008471;ENSBTAG00000012335;ENSBTAG00000019979;ENSBTAG00000019616;ENSBTAG00000009933;ENSBTAG00000009768;ENSBTAG00000001292;ENSBTAG00000014707;ENSBTAG00000006694;ENSBTAG00000007881;ENSBTAG00000003366;ENSBTAG00000020166;ENSBTAG00000030913;ENSBTAG00000024874;ENSBTAG00000052306;ENSBTAG00000053806;ENSBTAG00000046580;ENSBTAG00000030932;ENSBTAG00000016061;ENSBTAG00000034349;ENSBTAG00000039861;ENSBTAG00000008909
response to stress (GO:0006950)	< 0.001	ENSBTAG00000008021;ENSBTAG00000008471;ENSBTAG00000012335;ENSBTAG00000019616;ENSBTAG00000009933;ENSBTAG00000009768;ENSBTAG00000001292;ENSBTAG00000014707;ENSBTAG00000006694;ENSBTAG00000007881;ENSBTAG00000003366;ENSBTAG00000020166;ENSBTAG00000030913;ENSBTAG00000052306;ENSBTAG00000053806;ENSBTAG00000046580;ENSBTAG00000030932;ENSBTAG00000016061;ENSBTAG00000039861;ENSBTAG00000008909
response to type I interferon (GO:0034340)	< 0.001	ENSBTAG00000030913;ENSBTAG00000052306;ENSBTAG00000014707;ENSBTAG00000039861;ENSBTAG00000007881
response to virus (GO:0009615)	< 0.001	ENSBTAG00000008021;ENSBTAG00000008471;ENSBTAG00000009768;ENSBTAG00000014707;ENSBTAG00000007881;ENSBTAG00000003366;ENSBTAG00000020166;ENSBTAG00000030913;ENSBTAG00000052306;ENSBTAG00000053806;ENSBTAG00000046580;ENSBTAG00000030932;ENSBTAG00000016061;ENSBTAG00000039861
synaptic vesicle budding (GO:0070142)	0.009	ENSBTAG00000030913;ENSBTAG00000008471
synaptic vesicle budding from presynaptic endocytic zone	0.007	ENSBTAG00000030913;ENSBTAG00000008471

membrane (GO:0016185)		

Supplementary Figures

Supplementary Figure 1. Multidimensional scaling plot with normalized values for prenatally stressed relative to control samples prior to outlier removal; BCV: biological coefficient of variation method

Supplementary Figure 2. Multidimensional scaling plot with normalized values for male relative to female samples prior to outlier removal; BCV: biological coefficient of variation method
